# Emerging Therapeutic Strategies for COVID-19 patients

**DOI:** 10.15190/d.2020.2

**Published:** 2020-03-12

**Authors:** Shudong Zhu, Xialing Guo, Kyla Geary, Dianzheng Zhang

**Affiliations:** Argus Pharmaceuticals, Changsha, China; Department of Bio-Medical Sciences, Philadelphia College of Osteopathic Medicine, Philadelphia, PA USA

**Keywords:** Coronavirus, 2019-nCoV, SARS-COV-2, COVID-19, remdesivir, chloroquine phosphate, abidol, lopinavir/ ritonavir, plasma.

## Abstract

Over 100,000 cases of COVID-19 patients infected with the novel coronavirus SARS-COV-2 have been reported worldwide in approximately 2 months, resulting in over 3000 deaths. Potential therapeutic strategies, including remdesivir, chloroquine phosphate, abidol, lopinavir/ritonavir, plasma, antibody, vaccine and stem cells are discussed in this review. With the number of patients increasing daily, there is an urgent need for effective therapeutic intervention.

## **1.** **Introduction**

Since the beginning of December 2019, numerous hospitals in Wuhan, China have reported an increasing number of patients with pneumonia due to the infection with an unknown virus. The virus, first named 2019 novel Corona Virus (2019-nCoV), was later changed to Severe Acute Respiratory Syndrome Corona Virus 2 (SARS-COV-2), and the pneumonia was named Corona Virus Disease 2019 (COVID-19). World Health Organization (WHO) declared COVID-19 a Public Health Emergency of International Concern on January 31, and, as of March 2020, the virus has infected >90,000 people, and killed >3,100 worldwide^1^. With the number of patients increasing daily, there is an urgent need for effective therapeutic intervention. In this article, we will discuss several therapeutic strategies against COVID-19 infection.

## **2.** **Nucleotide analogs**

**Figure 1:**
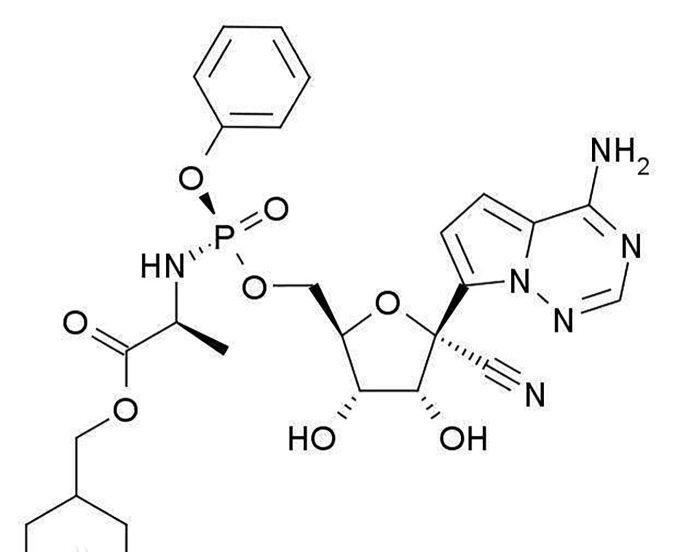
The structure of remdesivir, a nucleoside analog with antiviral activity. Results from experiments conducted on Vero E6 cells indicate that the EC90 for SARS-COV-2 is 1.76μM^4^.

The first case of COVID-19 in the United States was confirmed on January 20, 2020, by qPCR assay with samples from the patient’s nasopharyngeal and oropharyngeal swabs. Probes were based on genomic sequence of 2019-nCoV released by scientists from China. After the development of radiographic pneumonia, the patient was treated with remdesivir intravenously and almost all clinical symptoms resolved the following day^2^. Remdesivir (**Figure 1**) is a nucleotide analog originally developed by Gilead as a drug against Ebola virus. Mechanistically, it inhibits RNA-dependent RNA synthetase (RdRp) with completed Phase I and/or II clinical trials^3,4^. Therefore, Gilead has been working with China since February 2020 to conduct a phase 3 randomized, double-blinded, placebo-controlled, multicenter trial to determine the safety and efficacy of remdesivir on hospitalized patients with severe COVID-19^5^. The treatment was designed as 200 mg of remdesivir on day 1 followed by 100 mg/day for 9 days. The study is expected to be completed by May 1, 2020^6^. In addition, other antiviral nucleotide analogs such as fapivir and ribavirin are undergoing clinical trials. Fapivir is a drug approved in Japan for flu treatment.

## 3**. Chloroquine phosphate**

Chloroquine phosphate is an antimalarial drug. By increasing the pH of the nucleus, it can inhibit both the virus-host cell interaction and the pH-dependent virus replication^7^. The results from *in vitro* studies suggest that chloroquine can actively inhibit SARS-COV-2^4^. Both animal experiments and clinical trials are currently in progress. Results from preliminary clinical trials show that chloroquine phosphate has promising effects on patients with COVID-19. Of note, it has been reported that chloroquine may cause adverse reactions such as ocular disorders, immune system disorders, ear and labyrinth disorders, cardiac disorders, etc.^8^, therefore precautions and further testing are needed for the safe and effective treatment in COVID-19 patients.

## 4**. Abidol**

Abidol is a broad-spectrum antiviral drug. By inhibiting the fusion between the influenza virus and host cells, it can inhibit virus replication. It has been used to prevent/treat both SARS and Middle East Respiratory Syndrome (MERS). According to an online report, Abidol in the concentration range of 10-30μM not only inhibited COVID-19 duplication, it also significantly reduced the pathological effect of the virus^9^. A randomized multi-center controlled clinical trial wtih Abidol in patients with COVID-19 has started in Xiangya Hospital in China, which has been registered in the US clinical trial database^10^.

## 5**. Lopinavir/ritonavir **

Lopinavir/ritonavir is a combination of drugs mainly used for AIDS treatment. Lopinavir inhibits viral protease resulting in immature/non-infectious virus particles; ritonavir inhibits the degradation of lopinavir in the liver and thereby extends lopinavir’s half-life. Results from *in vitro* studies showed that lopinavir/ritonavir can inhibit the replication of both MERS and SARS^11^. However, whether they can inhibit COVID-19 is unknown. Therefore, a clinical trial to use lopinavir/ritonavir for COVID-19 treatment will be launched soon in a hospital in Wuhan, China. In addition, since darunavir (trade name: Prozekal) is another protease inhibitor used for HIV treatment, a combination of darunavir and ritonavir could also be a potential treatment of COVID-19, especially since darunavir has been approved in China since 2018 for HIV treatment.

## 6**. Plasma**

The company CNBG claimed on February 13, 2020, that plasma from recovered patients was used to successfully cure 11 critically ill patients with COVID-19^12^. The donated plasma with high-titer of SARS-COV-2 antibodies was confirmed without pathogens and with virus inactivation^12^. Of note, 12 to 24 hours post-treatment, the major inflammation symptoms decreased significantly with increased lymphocytes counts and blood oxygen saturation. Improved vital signs were also observed. Currently, although the exact underlying mechanisms are unknown, it is reasonable to speculate that the antibodies may bind the virus and prevent virus-host cell interaction, and therefore prevent infection. In addition, NK cells and other immune cells may also be involved in clearing the virus by antibody-dependent cell-mediated cytotoxicity. However, the potential risks associated with plasma usage, including pathogen transmission and allergic reaction, should be considered, and therefore this strategy may only be applied for a clinical emergency following standardized procedures. On the other hand, specific immunoglobulin could be a better option for treating critically ill patients with SARS-COV-2 infection.

## 7**. Antibodies**

Specific neutralizing antibodies against viral surface proteins may bind and prevent the virus from entering the host epithelial cells and subsequently prevent virus amplification. On the other hand, non-neutralizing antibodies may bind the virus and activate the immune cells (mainly macrophages) to engulf and clear the virus. However, excessive activation of these non-specific immune cells may cause the release of a large number of pro-inflammatory factors leading to cytokine storm and sepsis-related death. Therefore, non-neutralizing antibodies play an antiviral role in the early stages but may cause lung damage at later stages. However, it usually takes an extended period of time to generate monoclonal antibodies for a new pathogen that can be used clinically.

Regeneron Pharmaceuticals is using VelociSuite technology with a humanized mouse immune system to rapidly develop innovative antibodies for the treatment of virus sepsis. This strategy has shown positive results in clinical trials using antibodies to treat Ebola^13^. The German company Inflarx has developed a monoclonal antibody against the human C5a molecule. By specifically binding and inhibiting C5a-mediated biological functions, including the release of neutrophil chemotaxis and intra-cellular lysozyme, the antibodies can limit excessive inflammation without inhibiting immune function. Clinical trials on this antibody are now underway in China to potentially reduce both lung damage and death caused by sepsis infection^14^. 

## 8**. Vaccines**

There are now at least four candidate vaccines against the sepsis that are under development. Clinical trials on the most promising vaccine candidates may start in three to four months. However, it usually takes more than a year for a vaccine to become clinically applicable^15^.

## 9**. Stem cells**

Due to the self-renewal and multi-directional differentiation capability of mesenchymal stem cells, they can be differentiated to produce multifunctional cells. In damaged lung tissues, stem cells may be able to replenish bronchial epithelial and endothelial cells after the clearance of viral infection. They may also stabilize the pulmonary micro blood vessel and alveolar epithelial cell barriers. These effects could enhance the patient's immunity, alleviate pneumonia, and prevent secondary infections. As of February 14, 2020, more than 4 such projects have been approved for the treatment of COVID-19 in China, and another one has applied to the U.S. Food and Drug Administration (FDA) for emergency approval^16^.

## 10**. Others**

Other clinical trials of treatment options are also on the agenda. A search with the word “COVID-19” in the China Clinical Trials Registry (http://www.chictr.org.cn) revealed that 218 clinical trials have been registered on the platform as of March 9. Dozens of clinical trials have also been registered on the international platform of clinical trials (https://clinicaltrials.gov/). Recently, as a key target for therapeutic intervention, the cryo-EM structure of the CoV spike protein in its trimer form has been elucidated, which may further contribute to therapeutic designs in the near future^17^. 

## 11**. Conclusion**

Currently, remdesivir appears to be the most promising drug for the treatment of pneumonia caused by COVID-19 pneumonia. Many other agents or strategies are being tested for the treatment of patients with COVID-19. However, double-blind, randomized controlled trials are required to investigate their safety and efficacy.

## ** **KEY POINTS**

**◊** **The recent COVID-19 disease is due to the infection with the new coronavirus, SARS-CoV-2, causing a pandemic and necessitates the development of effective therapeutics.**

**◊ Many clinical trials are initiated worldwide. Potential therapeutics, including nucleotide analogs，chloroquine phosphate, abidol, lopinavir/ritonavir，plasma antibodies，vaccines, stem cells are among the most important strategies to be developed.**
